# The Hazardous NEOs of the Future: Long-term Predictions under Uncertainty

**DOI:** 10.1007/s40295-026-00598-8

**Published:** 2026-07-07

**Authors:** Oscar Fuentes-Muñoz, Davide Farnocchia, Steven R. Chesley, Ryan S. Park

**Affiliations:** 1 Jet Propulsion Laboratory, California Institute of Technology, 4800 Oak Grove Dr, Pasadena, 91109 CA USA; 2 Politecnico di Milano, Department of Aerospace Science and Technology, Via La Masa 34, Milan, 20156 MI Italy

**Keywords:** Asteroids, Near-Earth Asteroids (NEAs), Potentially Hazardous Asteroids (PHAs), Planetary Defense, Uncertainty propagation, Space Situational Awareness

## Abstract

Asteroid impact monitoring systems search for potential collisions of near-Earth objects (NEOs) with the Earth over 100 years. A necessary condition for an impact is the intersection between the orbit of the asteroid and the orbit of the Earth. This condition is measured by the Minimum Orbit Intersection Distance (MOID), which can be computed reliably for longer periods of time to identify when an Earth impact is possible. As the orbit is propagated into the future, the uncertainty in position grows faster than the uncertainty in the MOID. If the MOID is low but the position along the orbit is unknown, we compute an analytical approximation of the frequency of close encounters for a given distance. The NEO population spreads widely in orbital uncertainty, which we consider by propagating multiple samples from the initial orbital uncertainty distribution. We demonstrate and validate the methodology for 99942 Apophis, whose MOID is secularly increasing at a slow rate that still allows for future deep encounters. We apply this methodology to the NEO population, and for a large fraction we rule out the crossing of Earth’s orbit in the next 1000 years. Otherwise, we rank NEOs in terms of how long their MOID will be low, long-term frequency of close encounters, and frequency relative to the background close encounter frequency for objects of similar size. These rankings identify NEOs that should be prioritized for future tracking and orbit refinement.

## Introduction

The frequency and effects of asteroid impacts range from events such as the Chelyabinsk meteor in 2013, which caused hundreds of injuries [1], to extremely infrequent globally destructive events such as the Chicxulub impact, which marked the end of the Cretateous period 66 Myr ago [2, 3]. Even though harmful impacts are rare, planetary defense efforts are necessary to characterize and mitigate the potential risks associated with these events.

Asteroid impacts and close approaches are also very valuable scientific opportunities. Predicting asteroid impacts has allowed the recovery of meteorites [4], while close encounters allow detailed observations such as radar astrometry and characterization [5]. Predicted close encounters such as the 2029 flyby of 99942 Apophis are of extraordinary relevance [6], as shown by the planned ground-based observation campaigns and in-situ exploration missions [7, 8]. Future surveys will discover tens of thousands of new NEOs [9–11], allowing for more scientific opportunities and a more complete catalog of asteroids of interest for planetary defense.

Impact monitoring systems compute impact probabilities for every newly discovered asteroid, typically for 100 years. These systems search for impactors using different techniques, often using the fact that the uncertainty stretches in the direction of motion. JPL’s Sentry system^1^ searches for impactors within the asteroid’s uncertainty region [12]. ESA’s NEOCC^2^ uses the Aegis software to compute impact probabilities for 100 years [13], similarly to the NEODyS system^3^. In some cases the analysis is extended to further than 100 years, such as for 101955 Bennu [14], 99942 Apophis [15], or 1950 DA [16, 17].

A low Earth MOID is a necessary condition for an impact. The MOID (Asteroid-Earth MOID in this work) varies over time with the evolving osculating elements of the NEO and the Earth [18]. The proper elements of the asteroid allow to predict crossings with the orbit of the Earth. However, mean-motion resonances in the inner Solar System can make this prediction more difficult [19]. Based on numerical integration, the PHA population (NEOs with Earth MOID<0.05 au and H<22, roughly 140 m) was analyzed without considering the uncertainty in their orbits, showing a flow of asteroids out of the PHA category in timescales of centuries [20]. For the km-sized NEO population, the long-term impact hazard was assessed considering uncertainties in MOID and using analytical estimates for the probability of collision [21]. Near-Earth asteroids greater than 1 km are generally brighter, which means that they are observed more frequently. Hence, their orbital uncertainties are small.

This paper aims to study smaller objects and analyze the full range of sizes and uncertainties of the known NEOs. We identify the periods of time in which the MOID is below a distance threshold. Using an analytical approximation, we estimate the probability of collision during these times [21]. The discovery of new NEOs is biased towards low-MOID NEOs, including the ones small in size, which are less hazardous in case of an impact. Thus, we contextualize the estimated probabilities with respect to the size-dependent background encounter probability.

This manuscript is structured as follows. We start by describing the methodology through a few examples of the orbital propagation and introduce the metrics for uncertainty and long-term hazard. Then, our main results consist in the evaluation of the NEO population. We first describe the initial orbit uncertainties and how they grow. Then, we identify NEOs having a low-MOID for extended intervals and analytically estimate close approach frequencies. We evalute in more detail the orbital history and future of 99942 Apophis and show an example of Mean Motion Resonance effects. Throughout this work we use the term Mean Motion Resonance to refer to commensurability of the orbital periods, as we do not use a dynamical definition based on planetary gravitational forces.

## Orbit Long-term Propagation and Analysis

We consider ∼36,600 near-Earth objects from the discovered NEO population, with orbital solutions from JPL’s SSD Small-Body Database^4^. We propagate their orbits using JPL’s small-body integrator [22] between the dates 2000-01-01 and 3000-01-01. The numerical integrator is based on an *N*-body model including Sun, planets, Pluto, Moon and 16 main-belt asteroids. We use ephemeris models DE441 and SB441-N16 respectively for planets and small-body perturbers [23, 24]^5^

When available, non-gravitational accelerations such as the Yarkovsky effect are included in the nominal solution on JPL’s SBDB with the corresponding $A_2$ parameter [15]. In those cases we include them in the dynamical model and uncertainty sampling. There are 501 objects with $A_2$ included, and the median $\sigma _a$ of objects with $A_2$ is $7\cdot 10^{-9}$ au.

### Uncertainty Metrics

The uncertainty in semi-major axis $\sigma _a$ is a strong indicator of the uncertainty in orbital position and its growth after long propagations. Another commonly used metric for the uncertainty in the orbit is the condition code *U*. The object’s condition code indicates the orbital uncertainty with an integer from 0 to 9^6^. This parameter corresponds to the rounded logarithm of the angular runoff uncertainty $r_{off}$ in arcsec:

1 $$\begin{aligned} r_{off} = ( e\sigma _{\tau } + 10\frac{\sigma _P}{P}) \frac{k_0}{P} \cdot 3600 \cdot 3 \end{aligned}$$

2 $$\begin{aligned} U = \left\lfloor 9\frac{\ln {r_{off}}}{\ln {648,000}} \right\rfloor + 1 \end{aligned}$$

where $\lfloor \cdot \rfloor$ is the floor operator, *e* is the orbital eccentricity, τ is the time of perihelion (days), *P* is the orbital period (days), $k_0$ is the Gaussian gravitational constant (degrees/day), and σs are the 1-σ Gaussian uncertainties associated with the corresponding variables. A condition code of 0 indicates asteroids with well determined orbits, in which $r_{off}<1$ arcsec. On the other hand, a condition code of 9 indicates large uncertainty in the position of the asteroid along the orbit.

**Fig. 1:**
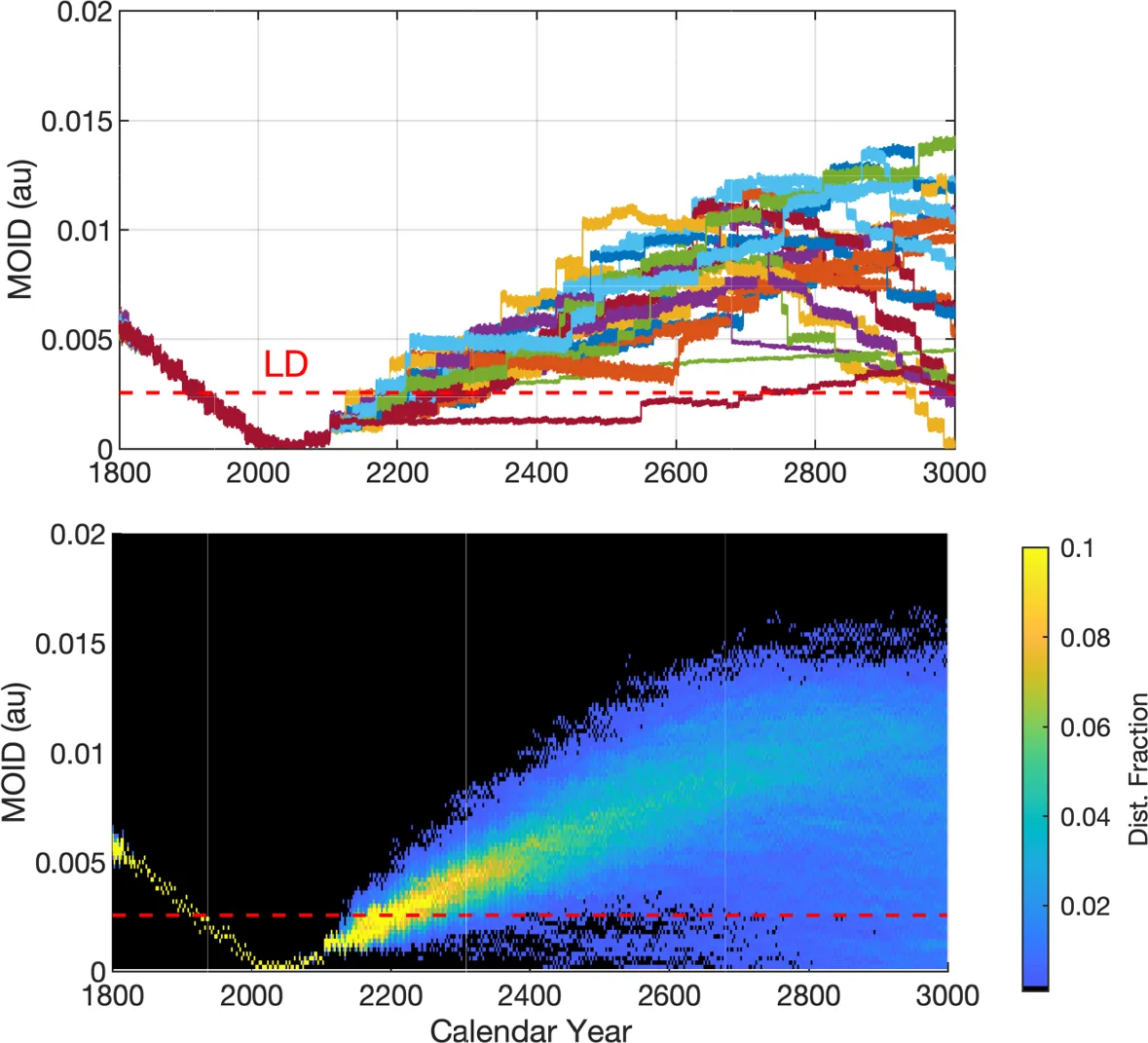
Evolution of the Earth MOID of 99942 Apophis from its current near-0 value to a secular increase of hundreds of years until the next near-0 MOID period. The top panel shows 20 runs (overlapping until past 2100) to illustrate the evolution of individual particles, and the bottom panel shows the statistical distribution of 1000 runs. The color intensity corresponds to the fraction of sampled particles in each region, saturated at 10% and abruptly blacked out in regions without samples to better highlight outliers and the diffusion of particles in MOID space. The red dashed line indicates a 1 Lunar Distance (LD) MOID threshold.

In general, we sample $N_{MC}=$ 24 test particles and the reference solution for each asteroid, based on the initial Gaussian uncertainty covariance and as a trade between the computational cost of studying the NEO population and the capability of tracking the growth in uncertainty in the MOID as necessary condition for close encounters. For objects highly ranked in hazard metrics, we increase this number to 96^7^. For validation in Section 2.2 we sample 1000 particles. We then propagate their orbits and compute the Earth MOID for each trajectory. Among the algorithms developed over the years to compute the MOID [25–27], we use the latter [27], considering its accuracy and computational speed. Notably, the other algorithms can be implemented with comparable performances. If the orbit of an NEO is well constrained, the MOID can typically be propagated with small uncertainty for thousands of years. 99942 Apophis is an illustrative counterexample, as shown in Figure 1. Even though the orbit is currently very well constrained, repeated close encounters with Earth cause an increase in the uncertainty within the next centuries. However, the probability of having a near-zero MOID after 200 years decreases for most of the remainder of the millennium. Overall, the Earth MOID of Apophis will remain relatively low, enough to maintain the classification of PHA (<0.05 au).

**Fig. 2:**
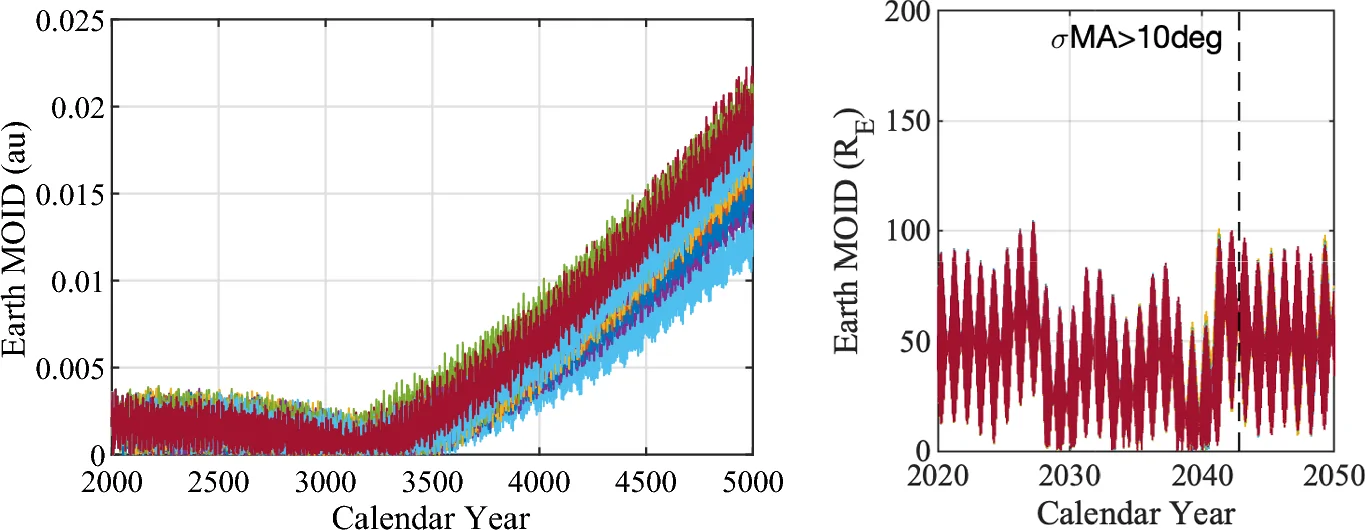
Earth MOID of 2023 VS3, a PHA of H=20.9 or approximately 250 m. The right panel shows the MOID in Earth radii in the next decades. The individual MOID evolution of 20 particles overlaps for most of the propagation. 2023 VS3 is an example of a large asteroid, with low-MOID for an extended period of time, and in which there is high uncertainty in the position: these are the objects we attempt to identify

Figure 2 shows an example of the propagation for 2023 VS3, which is found to have a low MOID for a longer than usual period of time. Given its initial large uncertainty with a condition code of 7, the position becomes hard to predict after a few decades. The standard deviation in mean anomaly exceeds 10 degrees in a few decades and 180 degrees by the 2220s. However, the MOID will most likely remain very low for at least 1500 more years, after which it secularly increases. The first goal of this study is to highlight objects with a low MOID for extensive periods of time, especially if the position becomes highly uncertain during this period. To measure this, we introduce the following metrics:

1. $year_{MA}$: The epoch at which the mean anomaly (MA) becomes unknown as a proxy for the orbital position uncertainty, which limits impact probability analyses. We determine it by the year in which standard deviation in the unwrapped^8^ mean anomaly σ> 180 deg.
2. $year_{MOID}$: The epoch at which the uncertainty in Earth MOID becomes large, which informs the long-term impact hazard characterization. We determine it by the year in which $\sigma _{MOID}>$1 LD (Lunar Distance).

**Fig. 3:**
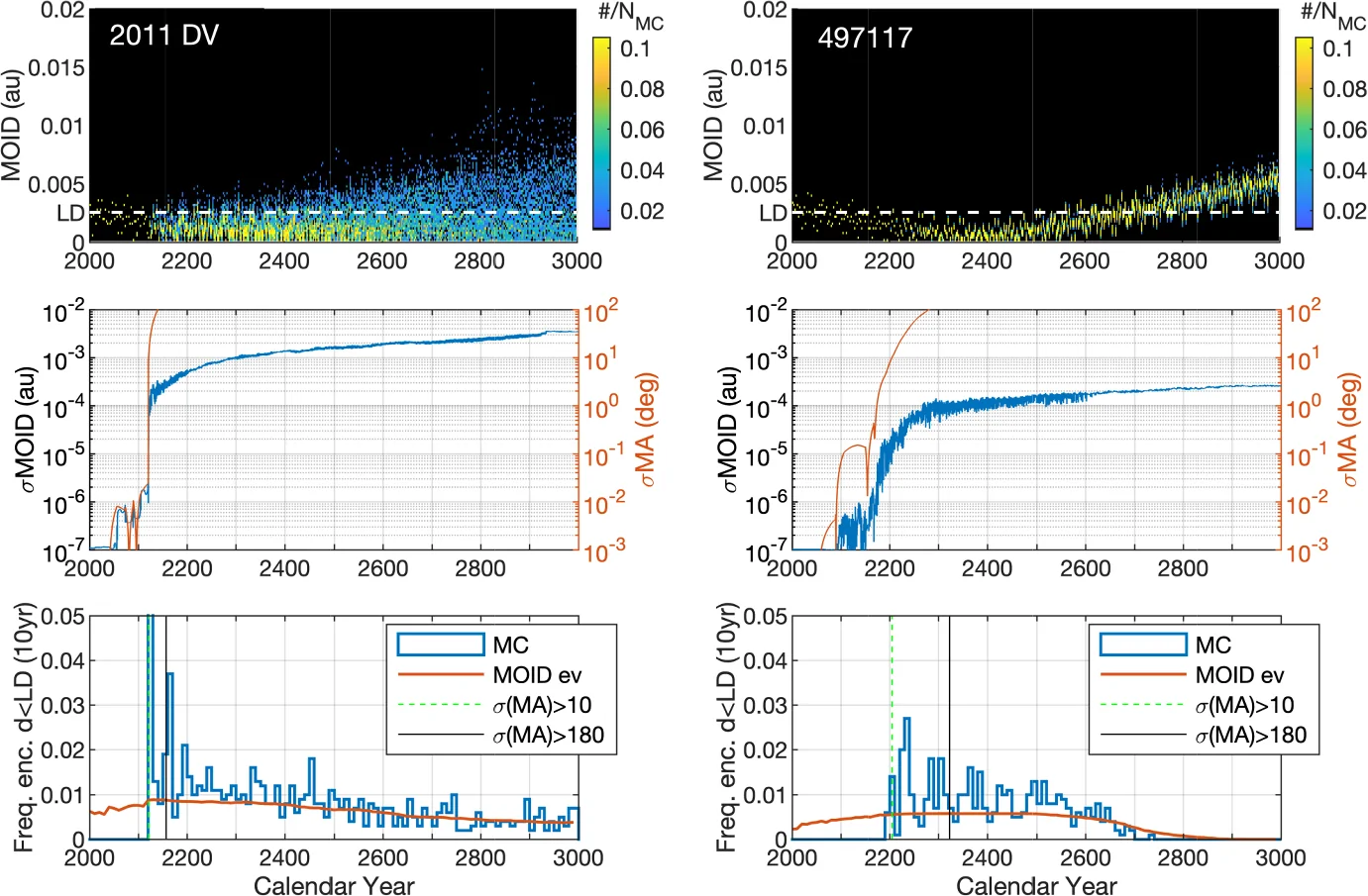
Demonstration of the MOID evolution, growth in uncertainty and how the metrics we introduce compare to statistics obtained from Monte Carlo sampling of the orbital uncertainty. (Top) Earth MOID evolution of asteroids 2011 DV and 497117, color coded as in Figure 1 for $N_{MC}=96$. (Middle) Increase in Earth MOID uncertainty and mean anomaly. (Bottom) Frequency of encounters of closest approach distance <1 LD in 10 years found with two methods: as statistics of the recorded encounters in a $N_{MC}=1000$ particle propagation, shown as a histogram; and as estimated from the MOID evolution of $N_{MC}=96$ particles, shown as the orange continuous line

Figure 3 introduces 2 more examples for discussion through the section, with the values of the metrics listed in Table 1. 2011 DV and 497117 are also found to have a low MOID for longer than usual periods of time. The increase in the orbital uncertainty of 2011 DV causes some of the samples to spread outside of the MOID< 1 LD region, whereas for 497117 the effect is an offset along the short-period oscillations of the MOID, without a spread. The middle row of Figure 3 shows how the uncertainty in the MOID of 497117 remains < 1 LD for the next thousand years, whereas $year_{MA}=2322$. The MOID evolution of 99942 Apophis described in Figure 1 can be quantified by $year_{MA}=$2164 and $year_{MOID}=$2634.

**Table 1:** Examples of the metrics introduced to characterize the long-term hazard risk of NEOs. U is the condition code, H is the absolute magnitude, $year_{MA}$ and $year_{MOID}$ describe growth in uncertainty as defined in Section 2.1, $F_{LD}$ describes how often the MOID is low, $P_{pre}$ is the frequency of encounters before $year_{MA}$, $P_{M}$ and $P_{CAT}$ are frequencies of encounters after $year_{MA}$ found by MOID propagation and in the close approach tables (more details in Section 2.2). *RR* is the risk relative to the background value, defined in Section 2.3

Asteroid	U	H	$year_{MA}$	$year_{MOID}$	$F_{LD}$	$P_{pre}$	$P_{M}$	$P_{CAT}$	$P_{Tot}$	*RR*
			$>180^{\circ }$	>LD						$\log {10}$
99942	0	19.1	2164	2634	0.25	1.11	0.16	0.06	1.27	-1.0
2023 VS3	7	20.9	2217	>3000	0.86	0.10	0.22	0.32	0.32	-2.1
2011 DV	0	20.7	2156	2794	0.71	1.04	0.56	0.64	1.60	-1.3
497117	0	18.3	2322	>3000	0.64	0.15	0.22	0.27	0.37	-1.3
143651	0	16.1	2747	2561	0.33	0.04	0.02	0.02	0.06	-1.1

### Long-term Hazard Metrics

During deep close encounters the orbit elements change abruptly, but the MOID remains nearly constant [18], rendering the MOID a more stable metric^9^. The next metric we introduce is the fraction of time from 2000-3000 in which the MOID is below a certain threshold: $F_{0.01}$ for MOID<0.01 au and $F_{LD}$ for MOID<1 LD. We average this fraction among the simulated samples of each NEO. Figure 1 shows Apophis’s Earth MOID is below 1 LD for $F_{LD}=$0.254 of the next thousand years, with most of the contribution in this and the next century, but back again toward the end of the millennium. In the cases of 2023 VS3, 2011 DV and 497117 (Figures 2 and 3) the fractions are respectively 0.86 (due to amplitude > LD), 0.71 (spread in trajectories) and 0.64 (secular increase).

We then focus on estimating the frequency of close encounters. Depending on the increase in positional uncertainty, we split the trajectory of a NEO in the following three phases:

1. Deterministic encounters: all the samples experience the same close encounters, and the uncertainty in time and closest approach distance is small. This is the realm of rigorous impact probability computation by monitoring systems.
2. Intermediate or transition: the samples are slightly spread along the orbit in a way that only a fraction of them may experience a close encounter. The closest approach distance and time is uncertain, and is often beyond the time limits set by impact monitoring systems.
3. Stochastic encounters: the samples are spread along the orbit and the mean anomaly is approximately uniformly distributed. If the MOID is low, we can estimate the frequency of encounters analytically, reducing the number of samples needed to capture the timing and capturing the spread in the overall orbit by the MOID.

We select the threshold beyond which the mean anomaly is uniformly distributed as the first time the standard deviation of the unwrapped mean anomaly is 180 deg. Thus, we consider the NEO is in the third phase after $year_{MA}$. Before this time, we track the close encounters that were found along the trajectories of the samples since 2025-01-01 and compute an average of $P_{pre}$ among the Monte Carlo samples. Previous works define a lower MA threshold of 10 degrees [21], allowing for the analytical estimate to represent a larger fraction of the trajectory. With this approach we combine the first two phases in $P_{pre}$. For the third, we use the following expression for the total frequency of encounters within a distance threshold *d* until the final epoch $t_{f}$:

3 $$\begin{aligned} P_{M} = \int _{year_{MA}}^{t_f}P_{MA}(d,K_E,K_{NEO})dt \end{aligned}$$

where $P_{M}$ is the frequency of encounters computed by *M*OID propagation, using this analytical approximation. $P_{MA}$ ($\hbox {yr}^{-1}$) is the probability in encounters per year assuming a uniform distribution in mean anomaly [28, 29], which is a function of the Keplerian elements of the Earth and the NEO ($K_E$ and $K_{NEO}$, respectively) and a distance threshold *d*. $P_{MA}$ is 0 if the MOID is bigger than the threshold *d*, which we find numerically over the simulation time. Final epoch $T_{S}$ is year 3000 throughout this work. In contrast to previous works [21], this expression is not normalized over the simulation time and thus the result is the cumulative number of expected close encounters. For this reason and by setting the threshold *d* to 1 LD, we are not computing impact probabilities, but rather the expected frequency of deep encounters of closest approach distance below 1 LD, which could be >1 in contrast to the natural upper bound to the frequency of collisions.

To show how this method compares to directly counting encounters during numerical propagation with higher $N_{MC}$, we also track the close encounters along the trajectory after $year_{MA}$. For the examples in this section, we set $N_{MC}=1000$ to have more reliable statistics comparing methods and show the results as $P_{CAT}$ in Table 1, which with this sample size corresponds to $\sim \pm 0.01$ uncertainty in $P_{CAT}$. The discrepancies between $P_{M}$ and $P_{CAT}$ are due to the assumptions on the mean anomaly, and show that $P_M$ is reliable in order of magnitude.

To gather the frequency of encounters throughout the simulation time, we define $P_{Tot}=P_{pre}+P_{M}$. In the examples of Table 1, we include two objects with $P_{pre}>1$ due to a close encounter certainly below 1 LD, this is the close encounter on 2120 of 2011 DV ($d_{CA}=0.386_{-0.005}^{+0.015}$ au) and, of course, the 2029 encounter of 99942 Apophis. 497117, whose MOID has an uncertainty <LD for centuries, allows us to discuss the $P_{pre}$ and the $year_{MA}$ threshold. Figure 3 shows that between σ(MA)>10 and σ(MA)>180 there are epochs of higher frequency of encounters than the estimate from a uniformly distributed assumption (labeled MOID ev). Thus, we are more conservative characterizing the close encounter frequency by setting the $year_{MA}$ threshold to σ(MA)>180 and introducing $P_{pre}$ until this point.

### Background risk

In general, the frequency of asteroid impacts has an inverse relationship to asteroid size, i.e., impacts of smaller asteroids are far more common. This distribution has been studied in the past, and allows us to contextualize the encounter frequencies with respect to the background impact frequency of close encounters. Thus, we can highlight NEOs that are rare in that their frequencies are larger than the background for objects of their absolute magnitude as a proxy for their size.

We estimate the background frequency using a previously derived recipe [30], partially reproduced here. The annual frequency *f* of encounters closer than a threshold distance *d* for objects of absolute magnitude smaller than *H* is found as follows:

4 $$\begin{aligned} f(d;H) = f(R_E,H) \left( \frac{d}{R_E} \right) ^2 \phi (d) \end{aligned}$$

where $f(R_E,H)=1.66\cdot 10^{-9}$ *N*(*H*) $\hbox {yr}^{-1}$ is the per-object impact frequency and *N*(*H*) is the number of near-Earth objects with absolute magnitude smaller than *H*. This distribution is tabulated [31] and can be approximated by the following polynomial fit in $\tilde{H}=$(*H*-20.250)/6.278 between H=9.5 and H=30.5:

5 $$\begin{aligned}  &   \log _{10}N(H) = 0.156 \tilde{H}^7 - 0.036 \tilde{H}^6 - 0.989 \tilde{H}^5 + 0.270 \tilde{H}^4 \nonumber \\  &   \quad + 1.974 \tilde{H}^3-0.160 \tilde{H}^2 + 1.584 \tilde{H} + 0.3788 \end{aligned}$$

where ϕ(d) accounts the gravitational focusing during a close approach. It is obtained by averaging over a statistical model of $V_\infty$ [30], leading to the following expression:

6 $$\begin{aligned} \phi (d) = \frac{1+\eta /d}{1+\eta /R_E} \end{aligned}$$

where η=2400 km. As *d* increases, ϕ(d) asymptotically approaches 0.73, and ϕ(d)=0.731 for d=1 LD. The relative risk is found as the ratio between probability we computed and the background frequency $RR=(P_{Tot}/\Delta t)/f(LD,H)$, with Δt being the 2025-3000 year period.

Among the example objects in Table 1, 143651 has the largest background-relative risk *RR*, close to Apophis with -1 largely due to the 2029 close encounter. We note that in the case of Apophis, *f*(38011 km$,19.09)=1.4\cdot 10^{-4}$, which corresponds to the frequently mentioned rarity of such encounters to occur every ∼7000 years.

With respect to the threshold of 1 LD, we expect encounters of PHA-sized (H<22) every ∼12 years and of km-sized (H<17.75) every ∼200 years. This is consistent with the 0 certain encounters for km-sized asteroids in the next 2 centuries and with the 7 encounters of discovered PHAs predicted/recovered in the 21st century: 99942 Apophis (Apr-2029), 2017 VW13 (Nov-2001), 308635 (Nov-2075), 2024 QP2 (Oct-2028), 153814 (Jun-2028), 308635 (Nov-2011), 2005 WY55 (May-2065).

## NEO Population Analysis

### Initial Orbital Uncertainties in NEO database

**Fig. 4:**
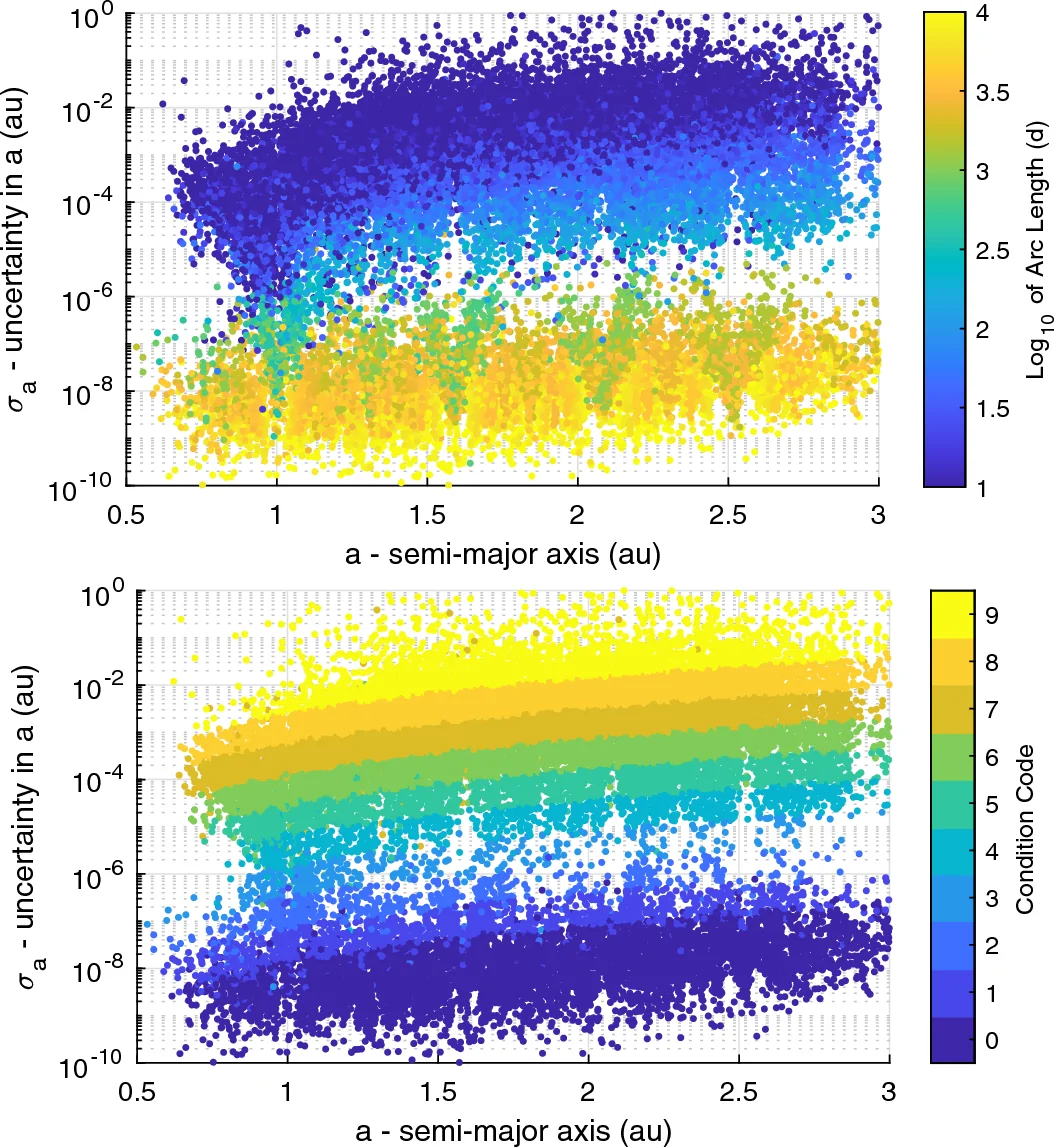
The distribution in orbital uncertainties of the NEO population can be understood by the observational arc length and orbital configuration. We show the uncertainty in semi-major axis as function of the semi-major axis. In the top panel the color code represents the length of the data arc in days, and in the bottom panel it represents the condition code

Figure 4 shows the semi-major axes of NEOs and their corresponding uncertainties. As the observational arc becomes longer, the uncertainty in the semi-major axis decreases by orders of magnitude. The distribution of uncertainties is thus clearly bimodal: asteroids observed for an arc shorter than a year, typically from a single apparition, have orders of magnitude larger uncertainty in their orbits. NEOs with semi-major axis near 1 au can have longer lasting apparitions and thus better determined orbits. If we split the two groups at $\sigma _a= 10^{-6}$ au, 73% of the NEOs have short observational arcs and relatively large uncertainties.

**Fig. 5:**
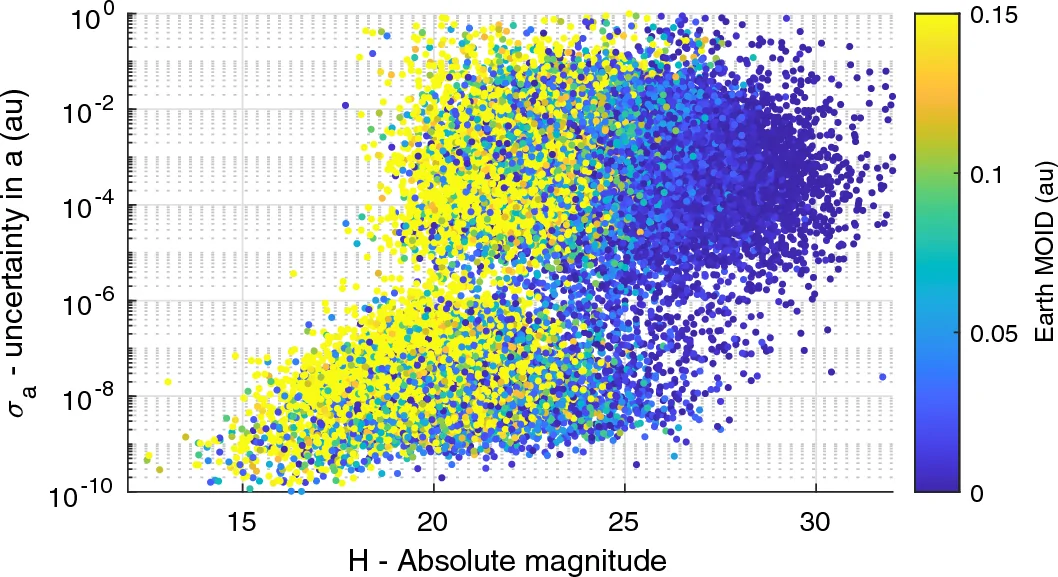
The orbital uncertainties of the NEO population are biased by observability: The size determines how far and often an object can be observed, while the Earth MOID how close can it get to the Earth. We show uncertainty in semi-major axis as function of the absolute magnitude. The color code represents the Earth MOID showing small objects can only be observed if they encounter the Earth at small distances

There is also a significant observational bias in the size distribution of the discovered NEO population. Figure 5 shows the uncertainties in semi-major axis as function of the Earth MOID and absolute magnitude *H*. *H* is a distance- and phase angle-independent measure of the asteroid brightness, inversely correlated to the asteroid size. For reference, the Chelyabinsk meteor was estimated to have a size of 17 to 20 m^10^, equivalent to approximately H=26.5. The first predicted asteroid impactor, 2008 TC3, had an estimated absolute magnitude of H=30.9 [4]. Small objects are more numerous and observable when they experience close Earth flybys, for which a low-MOID is a necessary condition. The overall Earth MOID distribution is skewed due to this effect: roughly 1 in 3 NEOs has an Earth MOID < 0.05 au and H>25 (roughly 35 m in diameter). The orbits of large NEOs are significantly better constrained. The median $\sigma _a$ of the 858 km-sized NEOs (H<17.75) is $4.9 \cdot 10^{-9}$ au. For the 10,989 objects of H<22, the median $\sigma _a=3.5\cdot 10^{-8}$ au. The overall median $\sigma _a$=$4\cdot 10^{-4}$ au is dominated by the majority of NEOs observed for short arcs.

Most of the km-sized asteroids have a condition code of 0 and thus their positions are currently well known. These small uncertainties allow reliable predictions of their orbits for thousands of years [21]. The PHA population is more evenly distributed among condition codes. Most of the NEOs with U≥7 correspond to smaller sizes, with absolute magnitudes around 25.

### Predictability: Orbital Uncertainty Evolution

**Fig. 6:**
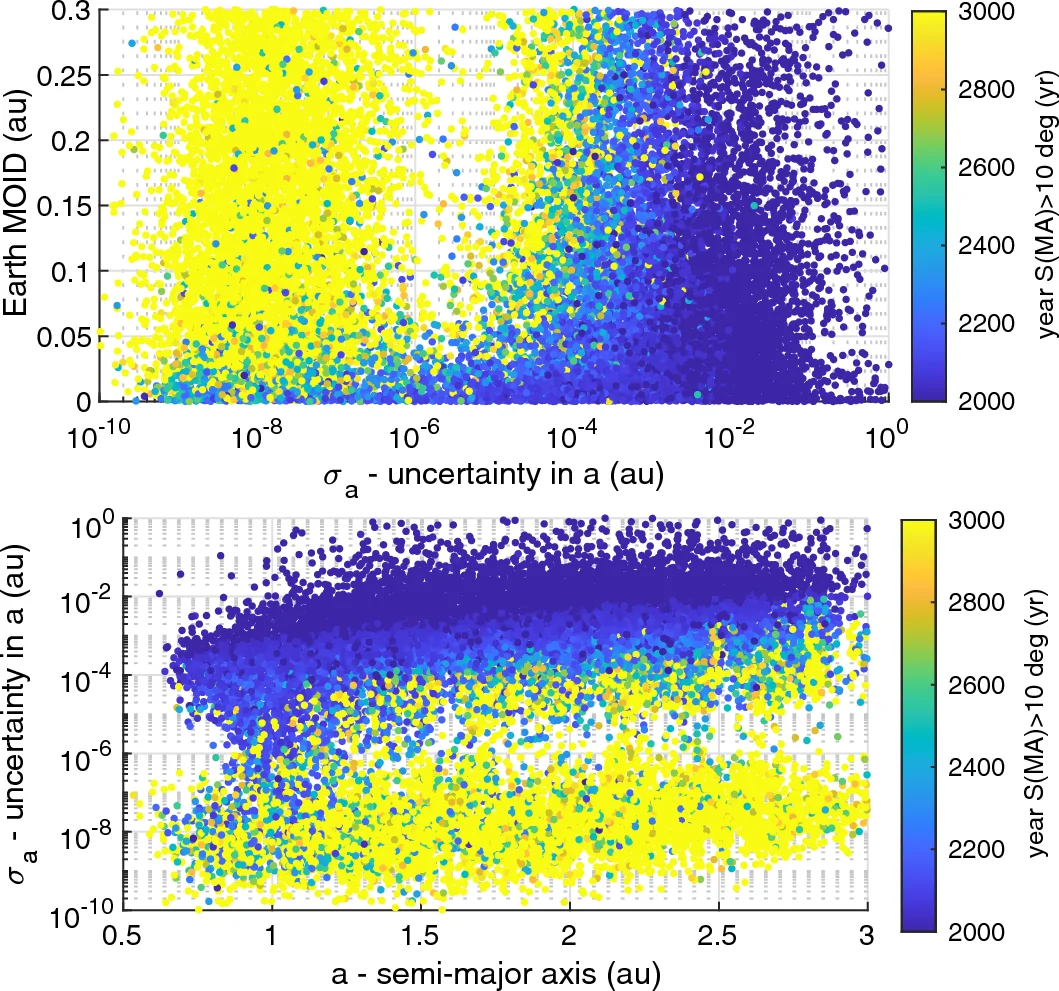
The current initial uncertainty has an influence in for how long we can predict the position, though the growth also depends on orbital region and close encounters. We show the time until the standard deviation in mean anomaly reaches 10 degrees as a function of MOID and semi-major axis uncertainty (top) and as function of semi-major axis and uncertainty in semi-major axis. (bottom)

This section describes the predictability of NEO orbits as function of the uncertainty in their current orbital state. In particular we use the two metrics we introduced in Section 2.1. Figure 6 shows $year_{MA}$ as function of *a*, Earth MOID, and $\sigma _a$. 95% of objects with $\sigma _a>10^{-6}$ au have $year_{MA}<$ 2100. Within the better observed group ($\sigma _a<10^{-6}$), $year_{MA}$ spreads between the next centuries to beyond the current simulation time. As we show in the examples of Section 2, it can be decades before a planet-encountering asteroid experiences the increase in $\sigma _a$ of a few orders of magnitude. This explains the earlier $year_{MA}$ for asteroids with currently a low-Earth MOID.

**Fig. 7:**
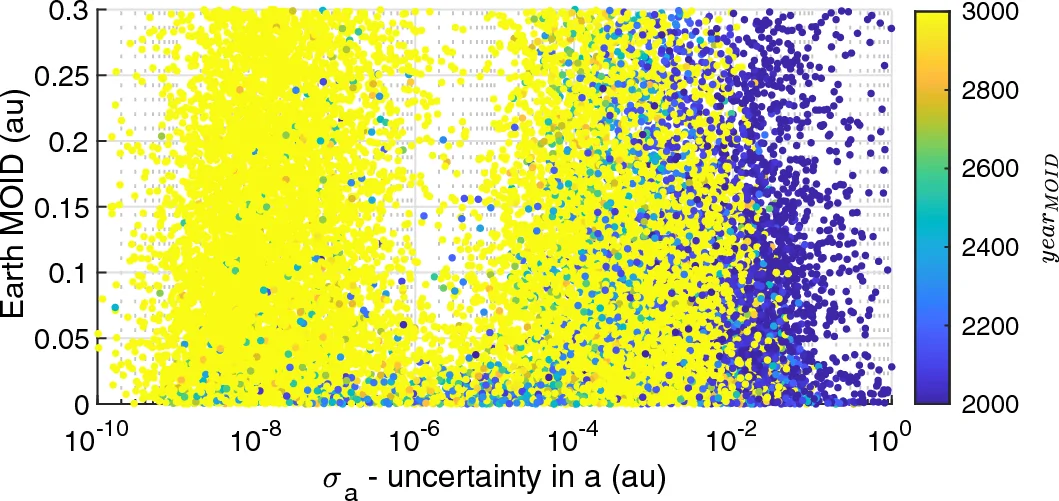
Time until the standard deviation in Earth MOID reaches 1 LD. In comparison to Figure 6, the MOID takes significantly longer periods of time to become stochastic

Most objects in the group with $\sigma _a<10^{-6}$ au remain with $\sigma _{MOID}<$ 1 LD until year 3000. This proves the MOID is a reliable metric to characterize the long-term impact hazard. Figure 7 shows $year_{MOID}$ as function of $\sigma _a$ and Earth MOID. An interesting region in Figure 7 is around $\sigma _a=10^{-6}$ au, mostly dominated by low-Earth MOID asteroids with a∼1 au (See Fig. 6), which also have a faster increase in $\sigma _{MOID}$.

### Long-term low-MOID NEOs

**Fig. 8:**
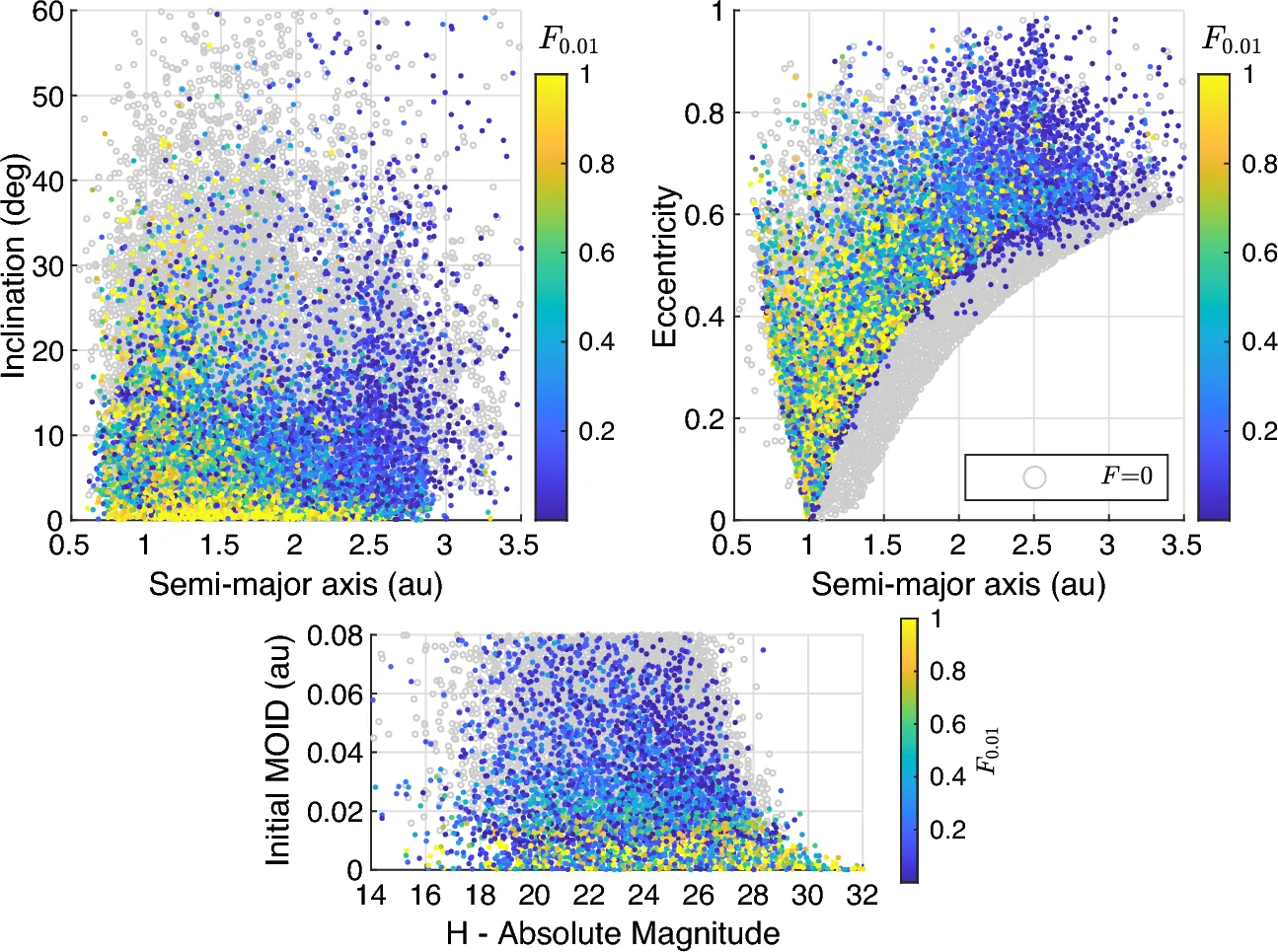
Depending on the orbit, asteroids spend more or less time in the vicinity of the Earth, which we show by the mean fraction of time with MOID<0.01, $F_{0.01}$. The panels show scatters of $F_{0.01}$ as function of semi-major axis, eccentricity and inclination (top) and as function of absolute magnitude and initial Earth MOID (bottom)

Figure 8 shows the fraction of time in which the Earth MOID remains below 0.01 au, $F_{0.01}$. As seen in the semi-major axis-eccentricity scatter, most of the objects with $F_{0.01}$ close to 1 are within the region bounded by q≤1 au and Q≥1 au, as these are conditions for an Earth-crossing orbit. Considering the NEO category is defined by q<1.3 au, there is a “band" region between q=1 au and q=1.3 au with few Earth encounters below 0.01 au (Top-right panel). Only about 6% of these NEOs are found to attain an Earth MOID < 0.01 au in the next 1000 yr, and most of those have a>2 au. Highly-inclined asteroids have a low MOID when their orbital nodes cross the orbit of the Earth. Thus, they have a low MOID for a shorter period of time. The best predictor for whether or not the MOID remains below a certain distance threshold is the MOID at the current epoch $\hbox {MOID}_0$. We find 120 asteroids with a MOID below 1 LD for 95% of the 2000-3000 year period. Among those, the maximum initial Earth MOID is 1.33 LD. Figure 8 shows how most of the objects with $F_{0.01}$ close to 1 initially have an Earth MOID < 0.02 au and H>22 (bottom panel). This reflects a clear observational bias in the MOID-*H* distribution is clear in detecting asteroids persistently with a low MOID. Table 2 shows those objects with H<22 and with highest fraction of time with a low MOID. They all remain below 0.01 au for the simulation time and below 1 LD for a large fraction of the time.

**Table 2:** Asteroids of H<22 with highest fraction of time of low MOID

Designation	$F_{LD}$	$F_{0.01au}$	$\hbox {MOID}_{0} (LD)$	H	U	a (au)	e	i (deg)
2023 GQ2	0.976	1.000	0.158	19.86	2	1.644	0.402	36.844
7482 (1994 PC1)	0.973	1.000	0.232	16.66	0	1.349	0.329	33.468
177049 (2003 EE16)	0.957	1.000	0.038	19.84	0	1.418	0.614	0.652
438908 (2009 XO)	0.946	0.994	0.714	20.63	0	1.859	0.543	0.349
529366 (2009 WM1)	0.919	1.000	0.088	20.42	0	1.181	0.169	25.767
620100 (2016 WJ1)	0.875	0.999	0.057	21.35	0	1.340	0.503	2.889
2023 VS3	0.859	1.000	0.796	20.90	7	1.914	0.487	24.200
2008 HB38	0.851	1.000	1.119	21.20	0	1.854	0.493	1.021
152685 (1998 MZ)	0.835	1.000	0.616	19.34	0	1.346	0.573	0.148
2018 GG2	0.834	1.000	1.245	19.26	0	2.236	0.710	43.316

**Fig. 9:**
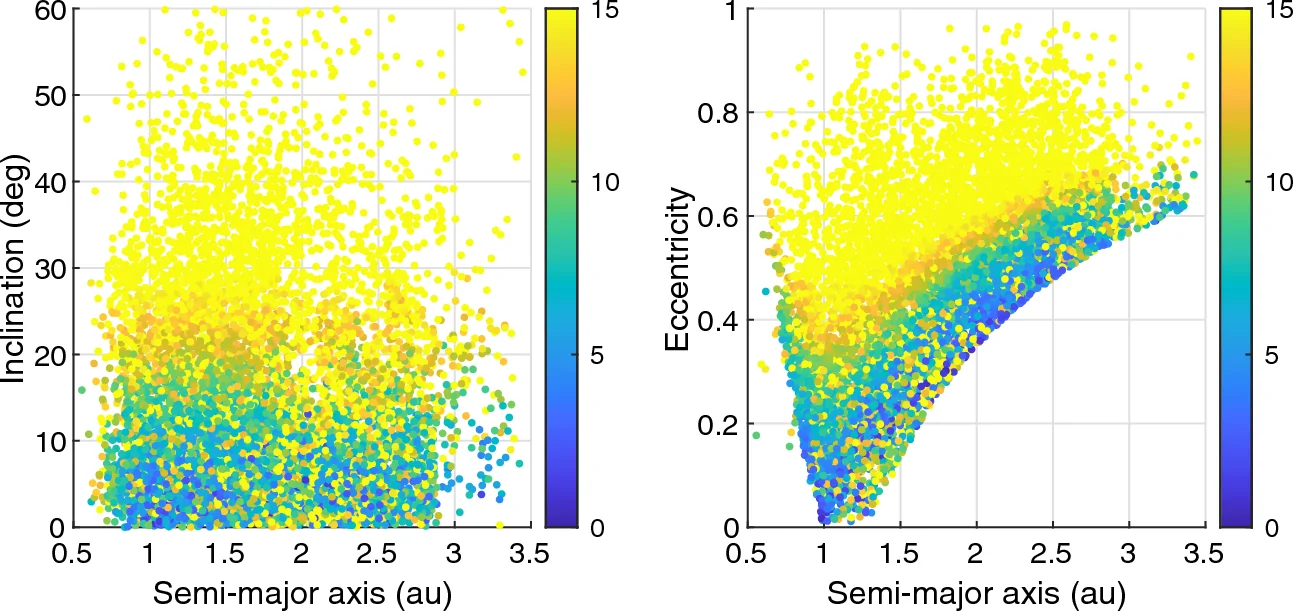
$V_\infty$ ($\hbox {kms}^{-1}$) at a hypothetical close approach with asteroid and Earth at the points that define the MOID, i.e., the closest possible encounter. The velocity of the flybys is implicit in the analytical metric of long-term hazard in equation 3 in $P_{MA}$

Computing the MOID requires finding the locations along the asteroid’s and Earth’s orbit that define the absolute minimum of the possible relative distances. At this configuration we compute the unperturbed velocity at infinity $V_\infty$, which is relevant for the estimation of the impact probabilities. Figure 9 shows the distribution of $V_\infty$ as a function of semi-major axis, eccentricity and inclination. As expected by analytical theories [18], $V_\infty$ increases with inclination and eccentricity.

### NEOs with higher Close Encounter Frequency

Tables 3, 4 and 5 show the highest ranked objects among the km-sized population (H<17.75), objects with H<22, and overall. The highest probabilities $P_{M}$ increase significantly as we relax the constraint on *H*.

We find that in most of the highly ranked km-sized asteroids the close encounters found before $year_{MA}$ contribute more than after, though not always. Considering PHA-sized asteroids and all sizes in Tables 4 and 5, in all cases $P_{M}>P_{pre}$ except for those with deterministic encounters.

**Table 3:** Km-sized NEOs with largest probability of having a close encounter below 1 LD

Designation	$P_{Tot}$	$P_{Pre}$	$P_{M}$	$F_{LD}$	years	$year_{MA}$	$\hbox {MOID}_{0}$	H	U
					<LD	$\sigma >180^{\circ }$	(LD)		
7482 (1994 PC1)	0.317	0.167	0.151	0.973	2000-3000	2691	0.23	16.66	0
175114 (2004 QQ)	0.094	0.094	0.000	0.046	2621-2669	2858	23.21	16.66	0
314082 Dryope	0.076	0.031	0.045	0.296	2263-3000	2626	6.16	17.50	0
5011 Ptah	0.065	0.042	0.024	0.149	2615-2826	2732	9.81	16.70	0
143651 (2003 QO104)	0.059	0.037	0.022	0.331	2000-3000	2747	0.58	16.09	0
214869 (2007 PA8)	0.056	0.052	0.004	0.093	2556-3000	2831	11.84	16.51	0
86819 (2000 GK137)	0.050	0.042	0.009	0.224	2419-2999	2754	6.53	17.47	0
4179 Toutatis	0.046	0.010	0.036	0.358	2410-3000	2739	2.53	15.30	0
20236 (1998 BZ7)	0.042	0.042	0.000	0.090	2782-3000	2994	19.54	17.70	0
90075 (2002 VU94)	0.010	0.010	0.000	0.088	2477-2641	2711	10.95	15.37	0
620095 (2016 CB194)	0.003	0.000	0.003	0.036	2018-3000	2745	0.09	17.70	0
508997 (2005 FL4)	0.002	0.000	0.002	0.010	2760-2890	2580	95.22	17.12	0
164121 (2003 YT1)	0.001	0.000	0.001	0.037	2000-3000	2581	1.79	16.17	0
2017 MK8	0.001	0.000	0.001	0.004	2923-3000	2608	66.85	16.84	1
614374 (2009 CR2)	0.001	0.000	0.001	0.005	2261-2997	2555	39.87	16.70	1
161989 (1978 CA)	0.001	0.000	0.001	0.002	2936-2999	2987	5.96	17.31	0
7092 (1992 LC)	0.001	0.000	0.001	0.038	2295-3000	2837	39.84	15.12	0
475665 (2006 VY13)	0.000	0.000	0.000	0.007	2462-3000	2432	54.96	17.22	0
85490 (1997 SE5)	0.000	0.000	0.000	0.002	2797-2916	2677	107.19	14.81	0
248590 (2006 CS)	0.000	0.000	0.000	0.005	2064-2995	2966	37.91	16.32	0

**Table 4:** Asteroids with H<22 with largest probability of having a close encounter below 1 LD. $\hbox {Asteroids}^{*}$ 2011 DV, 308635, and 99942 Apophis will have close encounters of $d_{CA}<$ 1 LD in April 2120, November 2075, and April 2029, respectively

Designation	$P_{Tot}$	$P_{Pre}$	$P_{M}$	$F_{LD}$	years	$year_{MA}$	$\hbox {MOID}_{0}$	H	U
					<LD	$\sigma >180^{\circ }$	(LD)		
2011 DV	1.598	$1.036^{*}$	0.562	0.714	2000-3000	2157	0.13	20.67	0
308635 (2005 YU55)	1.309	$1.021^{*}$	0.289	0.609	2000-3000	2172	0.17	21.91	0
99942 Apophis	1.272	$1.114^{*}$	0.158	0.254	2000-3000	2164	0.01	19.1	0
153814 (2001 WN5)	1.208	1.208	0.000	0.265	2000-2394	2371	0.58	18.29	0
530520 (2011 LT17)	1.208	1.094	0.114	0.382	2001-3000	2318	0.67	21.85	0
509821 (2008 WQ63)	1.137	1.031	0.106	0.517	2308-3000	2521	3.11	20.20	0
2015 PM	1.125	1.125	0.000	0.161	2374-2563	2658	5.39	20.48	0
456938 (2007 YV56)	1.081	1.031	0.050	0.313	2076-2645	2422	1.92	21.02	0
465824 (2010 FR)	1.032	0.979	0.053	0.300	2082-2582	2344	2.02	21.69	0
2011 JA	1.000	1.000	0.000	0.297	2000-2491	2749	0.80	21.44	0
153201 (2000 WO107)	1.000	1.000	0.000	0.193	2031-2238	2652	1.14	19.30	0
85640 (1998 OX4)	1.000	1.000	0.000	0.189	2000-2199	2499	0.40	21.14	0
2021 MK1	0.992	0.177	0.815	0.754	2000-3000	2155	0.95	21.32	2
509352 (2007 AG)	0.704	0.260	0.444	0.546	2000-3000	2557	1.79	20.16	0
415713 (1998 XX2)	0.587	0.000	0.587	0.282	2496-3000	2453	6.07	20.00	0
2009 BE58	0.585	0.042	0.544	0.656	2294-3000	2411	1.59	21.70	0
620100 (2016 WJ1)	0.571	0.219	0.352	0.875	2000-3000	2187	0.06	21.35	0
529366 (2009 WM1)	0.475	0.115	0.360	0.919	2000-3000	2345	0.09	20.42	0
549948 (2011 WL2)	0.468	0.281	0.187	0.469	2000-3000	2219	0.67	20.84	0
453563 (2010 BB)	0.421	0.000	0.421	0.332	2497-3000	2533	4.50	20.24	0

**Table 5:** NEOs with largest probability of having a close encounter below 1 LD. $\hbox {Asteroid}^{*}$ 2019 BE5 will have close encounters of $d_{CA}<$ 1 LD in January 2060 and January 2079.  indicates asteroids with non-zero impact probability in Sentry

Designation	$P_{Tot}$	$P_{Pre}$	$P_{M}$	$F_{LD}$	years	$year_{MA}$	$\hbox {MOID}_{0}$	H	U
					<LD	$\sigma >180^{\circ }$	(LD)		
2010 VC140	8.789	0.333	8.456	0.482	2000-3000	2161	0.93	27.90	7
2019 BE5	4.191	$2.427^{*}$	1.764	0.643	2000-3000	2292	0.04	25.10	2
2023 $\hbox {BZ3}^{\dagger }$	3.931	0.552	3.379	0.656	2000-3000	2197	0.40	29.08	7
2013 $\hbox {FU13}^{\dagger }$	3.475	0.677	2.798	0.566	2000-3000	2158	1.08	27.40	6
2009 $\hbox {OW6}^{\dagger }$	3.281	0.542	2.740	0.891	2000-3000	2205	0.64	25.40	7
2022 QX4	3.139	0.750	2.389	0.757	2000-3000	2183	0.02	24.70	2
2016 $\hbox {BV1}^{\dagger }$	2.876	0.531	2.345	0.549	2000-3000	2109	0.62	28.57	6
2019 $\hbox {XV}^{\dagger }$	2.684	0.083	2.600	0.316	2000-3000	2129	0.41	29.20	5
2024 HQ1	2.520	0.125	2.395	0.615	2012-3000	2197	1.24	28.34	6
2019 $\hbox {DG1}^{\dagger }$	2.485	0.135	2.350	0.294	2000-3000	2311	2.67	26.68	6
2024 BX1	2.169	2.000	0.169	0.168	2000-2235	2091	0.00	32.71	5
2019 $\hbox {CM5}^{\dagger }$	2.104	0.427	1.677	0.821	2000-3000	2195	0.39	27.85	7
2022 $\hbox {UL11}^{\dagger }$	1.899	0.833	1.066	0.278	2000-3000	2135	1.02	29.15	7
2024 $\hbox {AM4}^{\dagger }$	1.867	1.083	0.784	0.553	2000-3000	2099	0.03	28.71	5
2022 UC1	1.808	0.260	1.548	0.319	2000-3000	2202	1.75	26.41	5
2023 XJ2	1.765	1.083	0.682	0.529	2000-3000	2119	0.49	24.30	1
2024 TR4	1.732	1.083	0.648	0.929	2000-3000	2210	0.19	26.88	6
2001 AV43	1.714	1.125	0.589	0.746	2000-3000	2192	0.66	24.60	0
2007 BD	1.708	0.167	1.541	0.783	2000-3000	2167	1.29	25.40	6
2024 QH2	1.678	1.083	0.595	0.698	2000-3000	2199	0.39	26.87	7

### NEOs with higher Risk relative to Background

Based on *RR*, the metric for the risk relative to the background size-dependent frequency of encounters, we rank the NEO population. The top-10 ranked objects are in Table 6. Most of them are km-sized asteroids with a low $P_M$, though 3 PHA-sized objects are also in the list with higher $P_{Tot}$.

**Table 6:** Asteroids with largest relative risk

Designation	$\hbox {Log}_{10}$(*RR*)	$P_{Tot}$ ($\hbox {yr}^{-1}$)	$F_{LD}$	$\hbox {year}_{MA}$	$\hbox {MOID}_{0} (LD)$	H	U
7482 (1994 PC1)	-0.69	3.17$\cdot 10^{-1}$	0.973	2691	0.23	16.66	0
4179 Toutatis	-0.82	4.63$\cdot 10^{-2}$	0.358	2739	2.53	15.30	0
99942 Apophis	-1.01	1.27	0.254	2164	0.01	19.1	0
143651 (2003 QO104)	-1.12	5.89$\cdot 10^{-2}$	0.331	2747	0.58	16.09	0
497117 (2004 FU4)	-1.31	$3.67 \cdot 10^{-1}$	0.644	2322	0.99	18.33	0
2011 DV	-1.32	1.60	0.714	2157	0.13	20.67	0
5011 Ptah	-1.39	6.54$\cdot 10^{-2}$	0.149	2732	9.81	16.70	0
90075 (2002 VU94)	-1.50	1.04$\cdot 10^{-2}$	0.088	2711	10.95	15.37	0
509352 (2007 AG)	-1.56	7.04$\cdot 10^{-1}$	0.546	2557	1.79	20.16	0
152685 (1998 MZ)	-1.59	4.03$\cdot 10^{-1}$	0.835	2333	0.62	19.34	0

## Detailed Case Studies

### 99942 Apophis past and future

The close encounter of 2029 of 99942 Apophis may alter its physical properties in ways that exploration missions such as ESA’s RAMSES [8] or NASA’s OSIRIS-APEX [7] may be able to reveal. Thus, we sample a larger number of trajectories ($N_{MC}=10,000$) for a longer period of time into the past and into the future to understand the history of close approaches of 99942 Apophis.

**Fig. 10:**
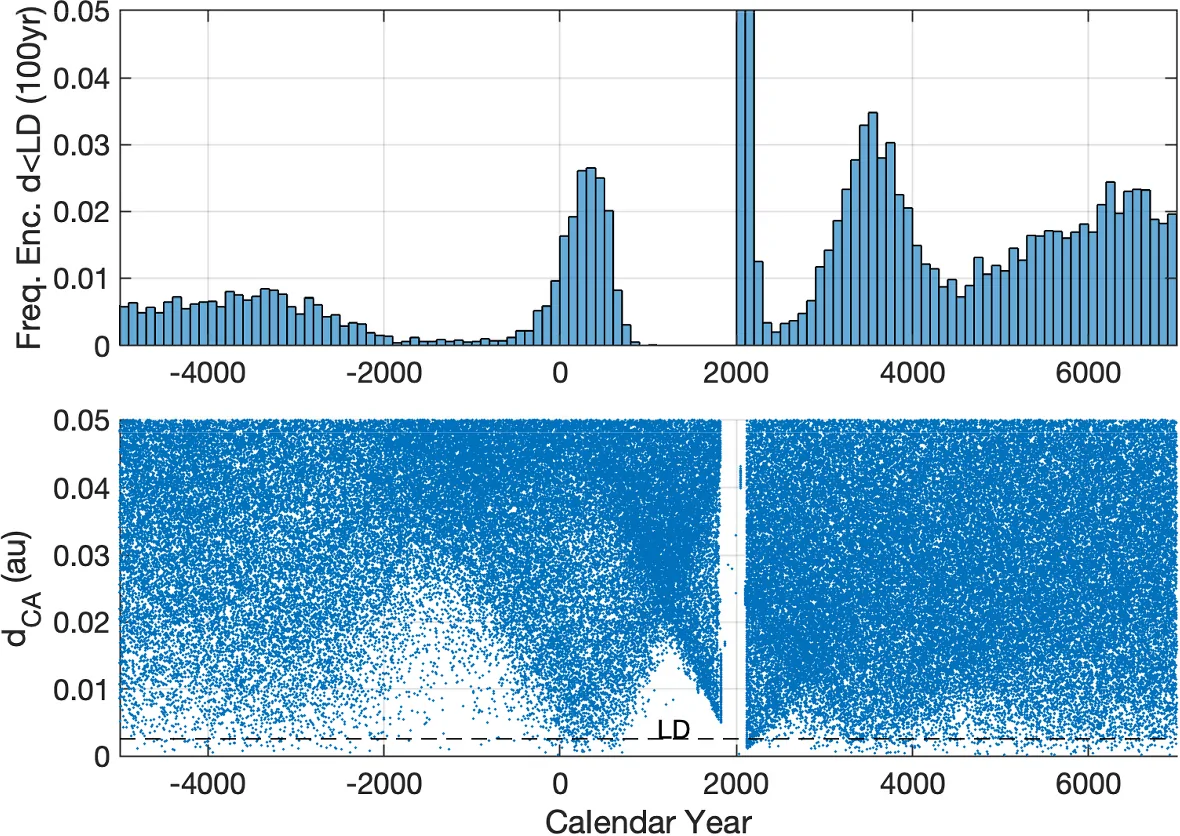
Frequency of close encounters in the past and future of 99942 Apophis. The top panel shows a histogram of the total encounters of $d_{CA}<$1 LD, whereas the bottom panel shows close encounters as a function of $d_{CA}$

We record all the planetary close encounters in the last 10,000 years between 99942 Apophis and Earth, Venus, and the Moon. The resulting dataset consists of  12 million planetary close encounters below 0.1 au between year 2025 and year -8000. We compute an equivalent propagation into the future for reference, unused for the statistical analysis. Figure 10 shows a subset of the distances of closest approach ($d_{CA}$) as a function of time. It also shows the deterministic part of the trajectory around the year 2000, i.e., where all the samples experience the same close encounters. Sometime between years -100 and 700 the MOID is near-zero, allowing again for deep Earth encounters.

**Fig. 11:**
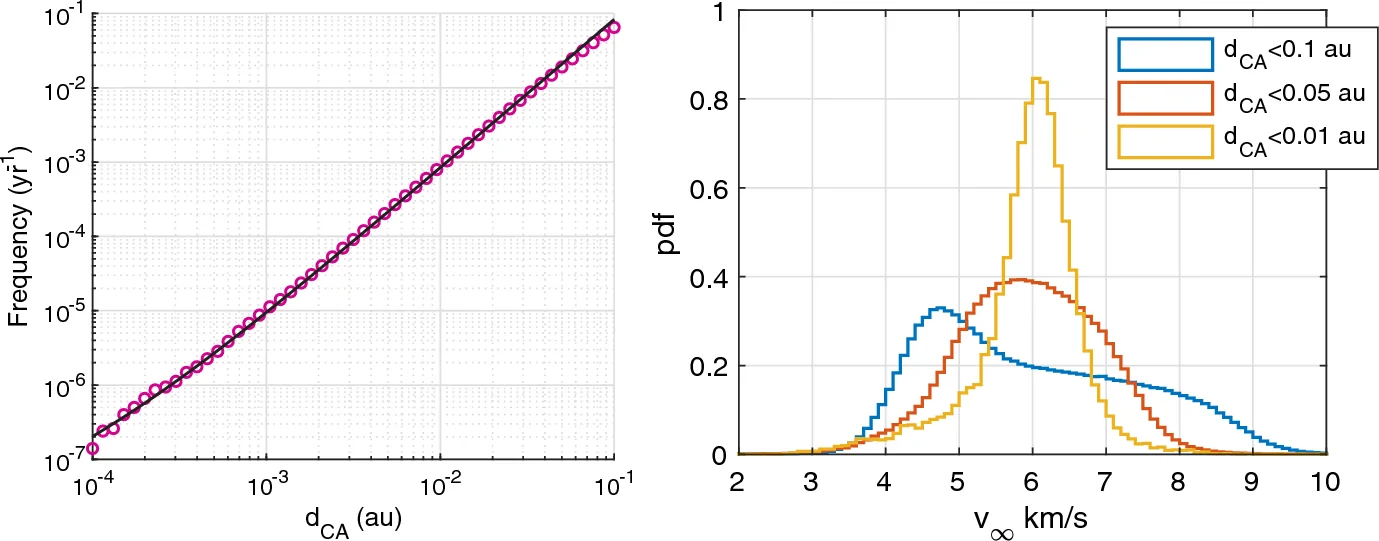
(Left) Statistical model of the close approaches below a distance threshold $d_{CA}$. The scatter represents the empirical model from numerical integration shown in Figure 10 and the line is the analytical fit of equation 8. (Right) Statistical distribution of $v_{\infty }$ depending on which distance threshold

Beyond year -3000 Earth close encounters become stochastic and more uniformly spread in time. In this region (years -8000 to -3000) we define a statistical model shown in Figure 11. We compute the frequency of encounters in this period as function of various distance thresholds and fit them to an analytical expression. We use non-linear least squares fit of the log10 of both frequencies and $d_{CA}$. The frequency of encounters of below a certain $d_{CA}$ threshold $P(<d_{CA}) \propto B_{\infty }^2$, where the impact parameter $B_{\infty }$ is related to $d_{CA}$ as [32]:

7 $$\begin{aligned} B_{\infty }^2=d_{CA}^2+(2GM_P/v_{\infty }^2) d_{CA} \end{aligned}$$

In this case, $v_{\infty }$ is also stochastic. Thus, we fit the probability of encounters $P(d_{CA}<d)$ to two parameters as $P=Ad_{CA}^2+Bd_{CA}$. *P* is in $\hbox {yr}^{-1}$, $d_{CA}$ in au, *A* in $\hbox {yr}^{-1}$ $\hbox {au}^{-2}$, *B* in $\hbox {yr}^{-1}$ $\hbox {au}^{-1}$. For close encounters of 99942 Apophis with Earth, we find A=8.3 $\hbox {yr}^{-1}$ $\hbox {au}^{-2}$ and B=0.0012 $\hbox {yr}^{-1}$ $\hbox {au}^{-1}$:

8 $$\begin{aligned} P_E (<d_{CA}) \simeq 8.3 d_{CA}^2+0.0012 d_{CA} \end{aligned}$$

The RMS of the fit in log10 is 0.045, meaning the empirical frequencies typically differ by a factor of ∼10% to the ones computed with the fitted analytical expression. We show the distribution of $v_{\infty }$ of Earth encounters in Figure 11, and find that the mean $v_{\infty }$ for Earth encounters of $d_{CA}<$ 0.05 au is 5.94 $\hbox {kms}^{-1}$. By setting a distance threshold of 0.1 au, we find close encounters spread along other locations in the orbit with a distribution that is less Gaussian.

At this point we can test if parameters *A* and *B* are consistent with equation 7, meaning $B/A\sim 2GM_p/v_\infty ^2$. First, we find that $2GM_p/v_\infty ^2=22,600$ km. Similarly, the ratio of the fit parameters is B/A=21,700 km. Table 7 shows the frequency and equivalent mean time between encounters for different thresholds of $d_{CA}$.

**Table 7:** Frequency and mean times between encounters in the history of 99942 Apophis. $R_L$ is the Roche Lobe assuming a density of 1.5 kg $\hbox {m}^{-3}$.

$d_{CA}$ (au)	Freq ($\hbox {yr}^{-1}$)	Mean time (yr)
0.1	0.08	12
0.01	8$\cdot 10^{-4}$	1200
LD=0.0026 au	6$\cdot 10^{-5}$	17000
$R_L$	4$\cdot 10^{-7}$	2.6$\cdot 10^{-6}$

The frequency of close encounters with Venus is fitted the close encounters found before year -3000. The resulting model is:

9 $$\begin{aligned} P_V (<d_{CA}) \simeq 8.3 d_{CA}^2+0.0012 d_{CA} \end{aligned}$$

The equivalent consistency test for the parameters of Venus yields $2GM_p/v_\infty ^2=29,000$ km and B/A=11,300 km, where the mean $v_\infty$ of the encounters below 0.05 au is 4.73 $\hbox {kms}^{-1}$. The fit is also slightly worse than for the Earth model, with an RMS in log10 of 0.06.

This analysis shows that with an extended integration we can build a statistical model of the frequency of encounters in an asteorid’s history, although with the caveat that we are extrapolating the uncertainty distribution of the period selected. When considering wether a close encounter perturbed Apophis’s properties, we shall consider both Earth and Venus encounters.

### Mean Motion Resonances

**Fig. 12:**
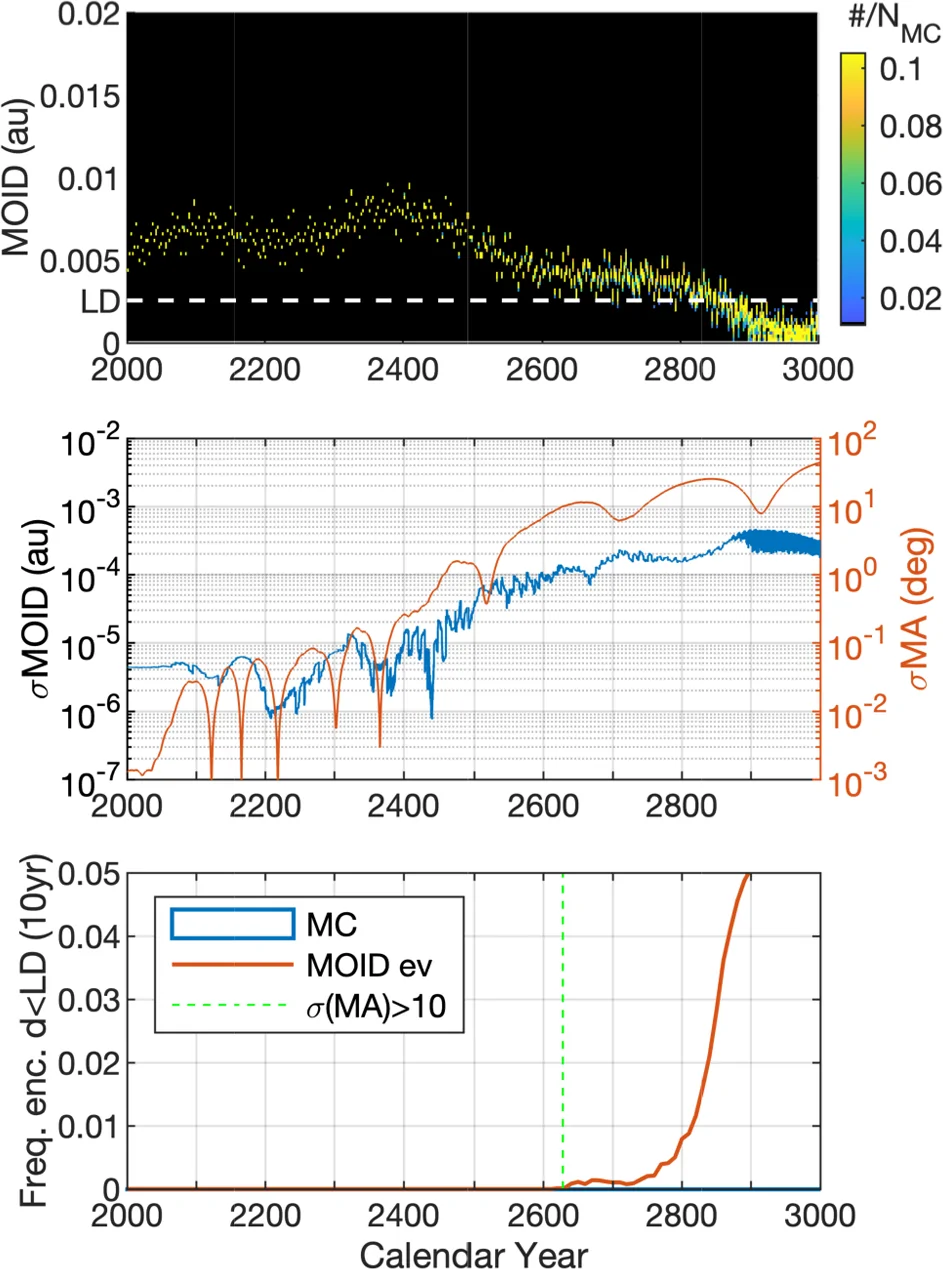
Earth MOID evolution and close encounters of asteroid 2021 MU2, formatted as Figure 3. 2021 MU2 is in resonance with the Earth, which in this case prevents it from having Earth encounters in the next millennium

The increased threshold in MA to define $year_{MA}$ allowed us to handle cases like 2021 MU2. Due to the low $v_\infty$ at the MOID location (aphelion distance Q=1.023 au), the analytical expression in equation 3 predicts high frequency of close encounters towards the end of the millennium. However, the simulations returned no close approaches with $d_{CA}<0.1$ au. Upon inspection, 2021 MU2 has an orbital period of 0.501 years and the phasing of the Earth resonance prevents it coming close to the Earth for extended periods of time. It also avoids close Venus encounters and its frequent Mercury encounters are not enough to spread the sampled trajectories. Despite some widening in the distribution of Mean Anomalies, these are still significantly far from being uniformly distributed. As a result, the bottom panel shows the absence of close encounters. Fig 12

These mean motion resonances helped the Earth be safe from this PHA within the time period of interest, but the opposite effect also occurs, asteroids can experience close encounters with a higher frequency than by assuming the position to be uniformly distributed along the orbit. In the case of resonances leading to consecutive encounters, these would occur before $year_{MA}$, which means that $P_{pre}$ should capture them.

## Conclusions

The NEO population is diverse in sizes and orbits, and as a result, in orbital uncertainties depending on how well they have been observed to date. We can clearly distinguish objects with long data arcs from objects observed for a single apparition, an observational window typically shorter than an orbital period. The latter group represents the majority of NEOs: smaller than PHAs and favoring lower Earth MOIDs as a result of the observational bias. NEO population models are developed from debiasing survey observations [33–35], we contribute to this discussion the resulting orbital uncertainties and implications for long-term orbital propagation. Whether or not the orbits of NEOs can be meaningfully predicted for long depends strongly on the dynamical regime. For instance, objects in Earth-like orbits are particularly sensitive to the initial conditions and may not be propagated deterministically for longer than ∼100 years, see for example the case of 2024 PT5 [36]. The stochastic approach of this work allows us to identify objects likely to remain in a very low Earth MOID orbit for extended periods of time. The expected frequency of deep encounters during those low-MOID periods allows us to rank asteroids on more hazardous orbits. For most km-sized asteroids and currently discovered PHAs, we can predict low-MOID periods during the next millennium. Most of the highly ranked asteroids with H<22 have an initial MOID<0.01 au, proving that the 0.05 au threshold is conservative in highlighting objects. Upcoming surveys will discover tens of thousands of new NEOs. In addition to population completeness goals, upcoming surveys will provide high observational sensitivity, follow-up apparitions, and potentially multi-apparition observational arches for newly discovered NEOs. These extended arcs will enhance our long-term hazard risk assessment capability.

Even after hazardous asteroid catalogs approach completeness, the necessary tracking should be established to prevent the natural growth in orbital uncertainty as we demonstrate in this work. Priority in tracking the increasingly large discovered NEO population may be granted using the metrics derived in this work, though the collision risk identified by impact monitoring systems shall take precedence. Impact monitoring systems target the following 100 years for impact hazard assessment. This work looks at longer time intervals and allows the identification of complementary sets of asteroids for further characterization. We highlight objects with encounters expected below 1 LD in longer timescales (typically smaller than PHAs and in uncertain orbits). A subset of those is composed by larger objects in well-determined orbits, though we do not find any with encounters more frequent than the background risk for their size.

## Data Availability

The dataset associated to this manuscript is available in the online version, including the 500 NEOs that were highlighted by the hazard metrics and evaluated with 96 samples each. In addition, any results with updated NEO population datasets may be accessed at the Zenodo dataset repository [37].

## Footnotes

1 JPL’s CNEOS Sentry System website.

2 ESA’s NEO SSA website.

3 NEODyS system website.

4 Publicly accessible at JPL’s Solar System Dynamics website, data retrieved 11-11-2024.

5 Report available at: https://ssd.jpl.nasa.gov/ftp/eph/small_bodies/asteroids_de441/SB441_IOM392R-21-005_perturbers.pdf

6 More information at the Minor Planet Center uncertainty parameter page

7 As multiple of the 12 cores available for computation closest to 100 runs.

8 Meaning the removal of >|π| radian jumps with $\pm 2\pi$ multiples to ensure angle wrapping does not create fictitious jumps in standard deviation.

9 With the exception of the rare heliocentric configuration allowing deep encounters with multiple planets in a short time span.

10 Reported in JPL press release of Feb 15, 2013
